# A case report of paraneoplastic bullous pemphigoid associated with mantle cell lymphoma: A rare presentation

**DOI:** 10.1097/MD.0000000000032822

**Published:** 2023-01-27

**Authors:** Ahmad Bakhtiar Md Radzi, Sazzli Shahlan Kasim

**Affiliations:** a Department of Cardiology, Faculty of Medicine, Universiti Teknologi MARA, Sungai Buloh Campus, Sungai Buloh, Selangor, Malaysia.

**Keywords:** bullous pemphigoid, mantle cell lymphoma, paraneoplastic

## Abstract

We report a rare case of paraneoplastic bullous pemphigoid associated with mantle cell lymphoma.

**Patients concerns:**

The patient presented with 5 months’ history of generalized skin itchiness, night sweat and loss of weight. The skin manifestations started over the foot and hand area. However, he started to developed tense blisters over the face, trunk and limbs 3 days prior to this admission.

**Diagnoses:**

The skin biopsy report showed subepidermal bullae, in which the immunofluorescence findings in keeping with bullous pemphigoid. The peripheral blood immunophenotyping was suggestive of mantle cell lymphoma. Hence, a diagnosis of paraneoplastic bullous pemphigoid associated with mantle cell lymphoma was made.

**Interventions:**

The patient was initiated with a cytoreduction chemotherapy.

**Outcomes:**

Unfortunately, patient’s condition deteriorated further due to neutropenic sepsis and he succumbed after 2 weeks of intensive care.

**Lessons:**

Bullous pemphigoid associated with mantle cell lymphoma are very rare. The presentation of bullous pemphigoid led to the detection of mantle cell lymphoma. Early diagnosis and appropriate treatment is crucial in managing this aggressive type of the disease. Both, bullous pemphigoid and mantle cell lymphoma had a parallel clinical course which suggests a paraneoplastic phenomenon in this reported case.

## 1. Introduction

Bullous pemphigoid is an autoimmune subepidermal blistering disorder. Mantle cell lymphoma is a B-cell malignancy of a rare type of non-Hodgkin lymphoma. Bullous pemphigoid associated with mantle cell lymphoma is even rarer which may suggest a paraneoplastic phenomenon.^[1]^

## 2. Case report

**Figure 1.**
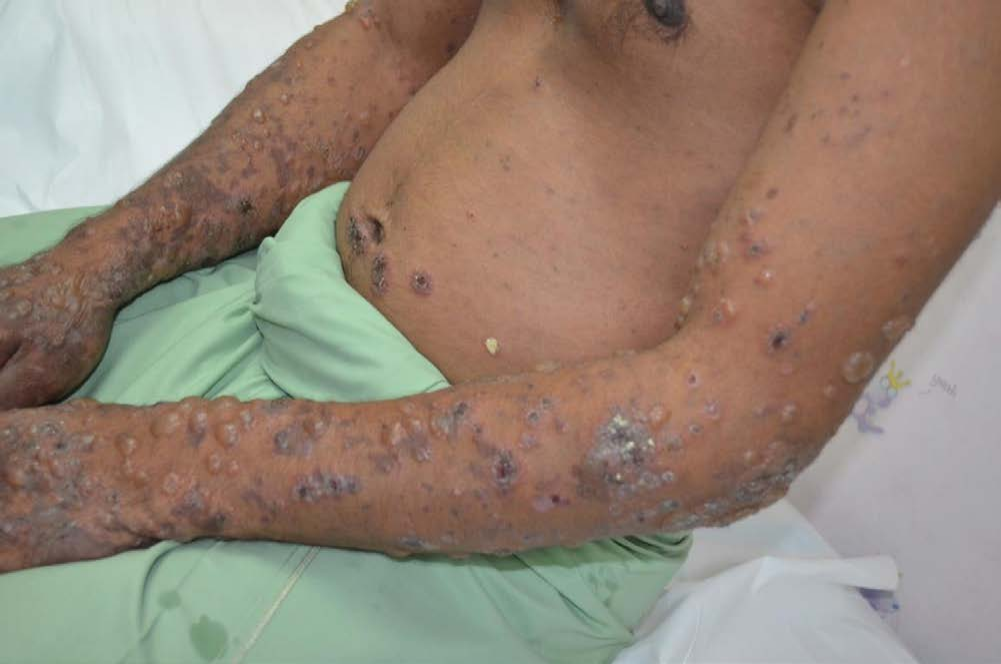
Multiple intact tense dome shape blisters with clear and hemorrhagic content, hemorrhagic crustings were found predominantly on the upper limbs and fewer on the anterior trunk and abdomen.

**Figure 2.**
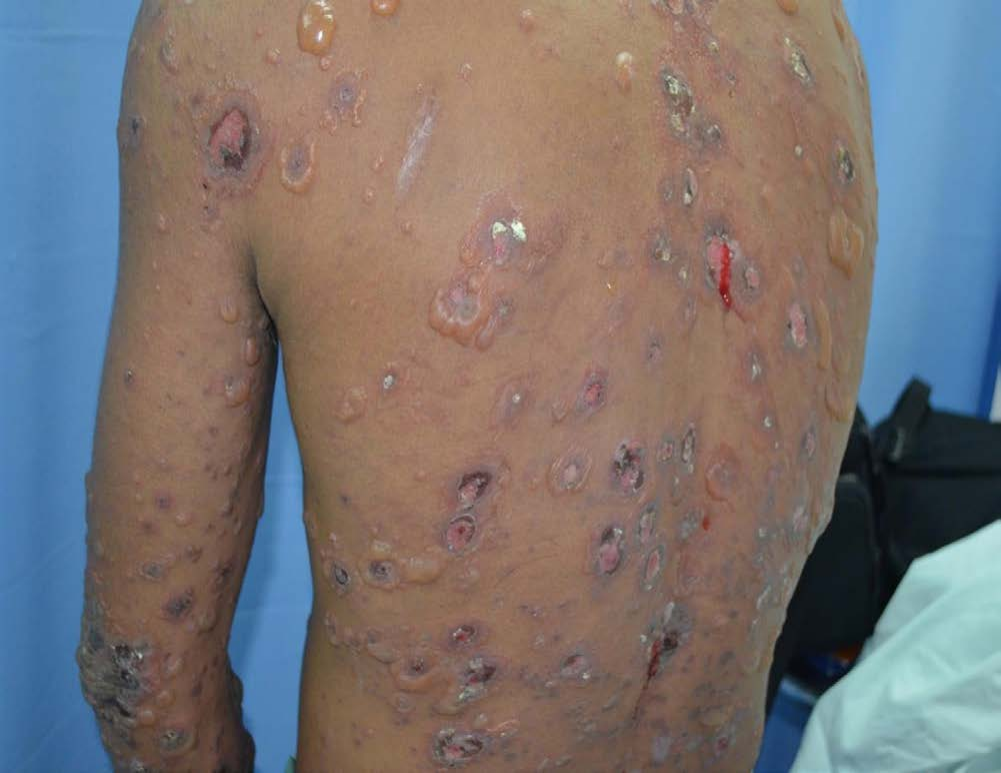
Multiple intact tense blisters, crusted erosions, and scarring on the patient’s back and upper limbs.

A 29-year-old Malay man, a federal plantation supervisor, was admitted with 5 months’ history of generalized skin itchiness, night sweat and loss of weight. The skin itchiness started over the foot and hand area. However, he started to developed tense blisters over the face, trunk and limbs 3 days prior to this admission. He gave history of applying sulfur-based powder solution on the itchy areas for 3 days’ duration prior to the blisters’ eruption. There was no prolonged fever, cough, recurrent oral ulcer, hair loss and joint pain. Family history was insignificant of malignancy or autoimmune disease. He neither had drug nor food allergy. Physical examination revealed multiple scaly and dry erosions on her scalp and crusted erosions on the face and periauricular region. Erosions were evidence on the hard palate and buccal mucosa. Multiple intact tense blisters with crusted erosions were found on the upper limbs, lower limbs, back, and lower abdomen (Figs. 1 and 2). The estimated body surface area involved was 10%. The Nikolsky sign was negative. Multiple shotty cervical, axillary and inguinal lymph nodes were palpable. Traube space was dull. Other systemic examinations were unremarkable.

**Figure 3.**
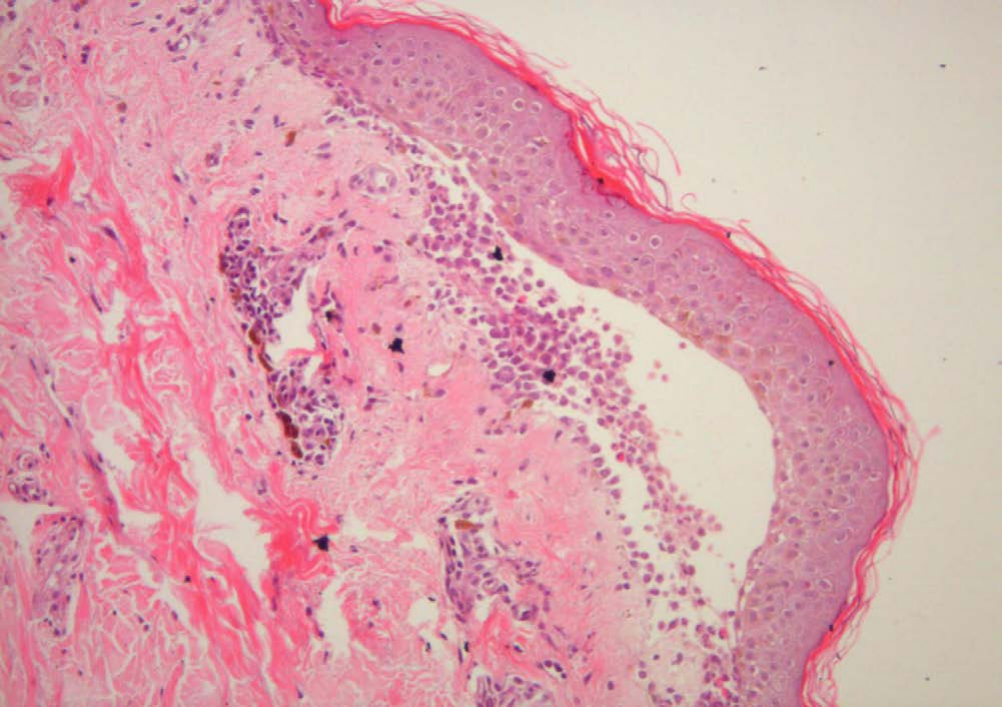
Skin biopsy showed subepidermal bullae, in which the lumen consists of inflammatory cells (H&E 40x magnification).

**Figure 4.**
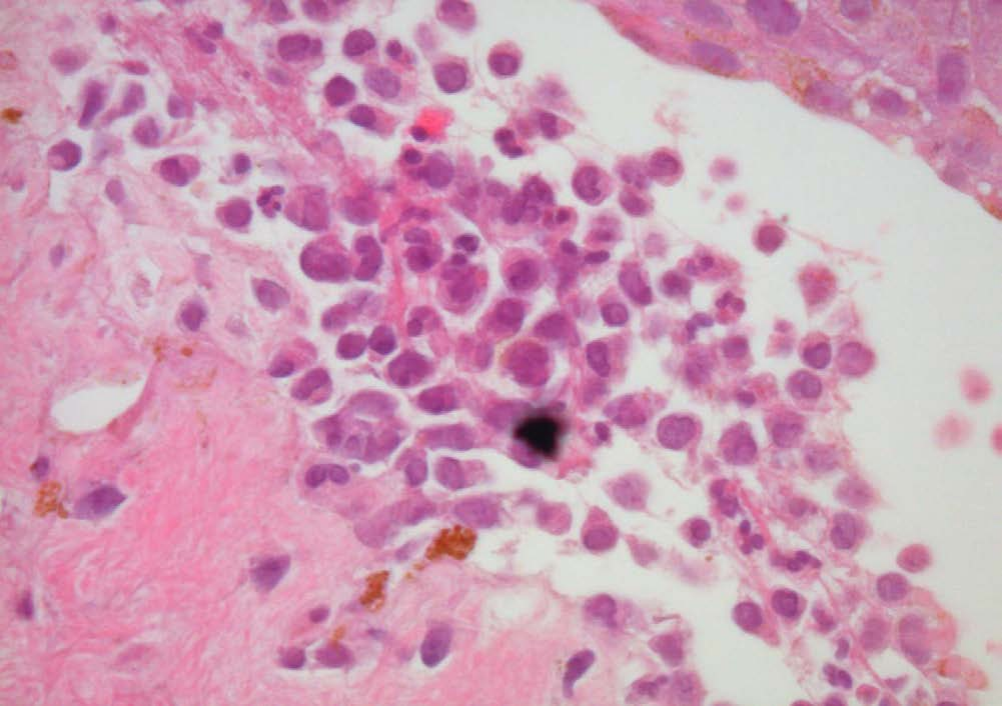
Skin biopsy showed subepidermal bullae, in which the lumen consists of acantholytic cells, neutrophils, and eosinophils (H&E 80x magnification).

The skin biopsy finding showed subepidermal bullae, in which the lumen consists of acantholytic cells and neutrophils, with positive immunofluorescence (IF) to immunoglobulin (Ig)-G, C3, C1q at the dermo-epidermal junction which in keeping with bullous pemphigoid (Figs. 3 and 4). There was no intra-epidermal or blood vessels immune depositions of IgA and IgM. Blood investigation results showed persistent leucocytosis (70–80 × 109/L) with predominant lymphocyte cells (90%). Full blood picture confirmed leucocytosis with moderate size abnormal lymphoid seen and indented nuclei. Peripheral blood for immunophenotyping showed presence of 71.7% abnormal cells expressing the following phenothypes; cluster of differentiation (CD)-45 bright/low side scatter, CD19 +ve, CD22 strongly +ve, CD20 +ve, CD5 +ve, FMC 7 +ve, CD 38 +ve, surface IgG IgM +ve, CD43 −ve, CD23 −ve CD25 −ve, CD103 −ve, and CD 11c–ve. There was lambda light chain restriction observed. The peripheral blood immunophenotyping was suggestive of CD5-positive B lymphoproliferative disease in keeping with mantle cell lymphoma. Hence, a diagnosis of paraneoplastic bullous pemphigoid associated with mantle cell lymphoma was made. Patient was started with intravenous hydrocortisone 100 mg 8-hourly followed by gradual tapering dose of oral prednisolone. Intravenous broad spectrum antibiotics were also administered. Gradual clinical improvements of his cutaneous lesions were observed after the course of treatment. In view of the diagnosis of mantle cell lymphoma, the patient was transferred to a tertiary hematology center. A bone marrow biopsy histopathological examination revealed infiltration by mantle cell lymphoma cells.

Patient was started with a cytoreduction chemotherapy regime comprised of rituximab, cyclophosphamide, doxorubicin hydrochloride, vincristine sulfate and prednisolone. Unfortunately, patient’s condition had deteriorated further due to neutropenic sepsis and patient succumbed after 2 weeks of intensive care.

## 3. Discussion

Our patient presented with bullous pemphigoid associated with mantle cell lymphoma. Bullous pemphigoid is an autoimmune subepidermal blistering disorder which occurs mainly in the elderly but may rarely present in children and younger adults. It is an acquired autoimmune disease which specifically involved the deposition of IgG and/or C3 along the basement membrane zone on direct IF. Circulating IgG autoantibodies are presence in most patients that can be demonstrated by indirect IF. Different types of malignant diseases can present with bullous pemphigoid, but it is still unclear with regards to the paraneoplastic significance of this association.^[2–4]^

A study showed that 11% of 110 cases of bullous pemphigoid were associated with carcinoma.^[1]^ Production of antibodies to tumor-specific antigens that might cross-react with the basal membrane zone would explain the relationship between carcinoma and bullous pemphigoid. Another theory suggested that secondary production of anti-basement membrane zone antibodies by the tumor cells occurred through secretion of a substance that could damage the basement membrane. Theory of genetic predisposition to both diseases or the possibility that the same external agent might generate the cancer and the basement membrane zone damage was explored.^[5]^ In B-cell neoplasia, the rare occurrence of autoantibody-mediated skin disease has been reported in association with paraneoplastic pemphigus.^[6,7]^

Mantle cell lymphoma is a B-cell malignancy of a rare type of non-Hodgkin lymphoma. It comprises 3 to 10% of all non-Hodgkin lymphomas.^[8]^ It is derived from a subset of naive pregerminal center cells that are localized in the mantle zones of secondary follicles or in the primary follicles. Chromosomal translocation t(11;14)(q13;q32) is characteristic of mantle cell lymphoma.^[9]^ Mantle cell lymphoma is commoner among older adults with a median age in their 60s and has a male predominance.^[10,11]^ Mantle cell lymphoma is diagnosed based on a biopsy of a lymph node, tissue, bone marrow or blood phenotype which shows the typical morphology of monomorphic small to medium sized lymphoid cells with irregular nuclear contours.^[12]^ Small cell, mantle zone, diffuse and blastic are four recognized mantle cell lymphoma cytologic variants.^[13]^ Immunophenotyping for mantle cell lymphoma is commonly positive with CD20, CD5, and cyclin D1 while being negative for CD10 and Bcl6.^[12]^

Mantle cell lymphoma has an aggressive clinical course with a pattern of resistant and relapsing disease making it incurable to standard therapy with a median survival rate of 4 to 5 years.^[14]^ Patients usually present at stage III/IV disease with extensive lymphadenopathy, blood and bone marrow involvement, and splenomegaly,^[15]^ as seen in our patient. Due to advanced stage disease with associated paraneoplasticbullous pemphigoid, the patient was started with cytoreduction regime consist of rituximab plus chemotherapy. Options of treatment include rituximab-fludarabine, cyclophosphamide, rituximab-cyclophosphamide, vincristine, prednisone, rituximab-cyclophosphamide, vincristine, doxorubicin, prednisone, rituximab-bendamustine, and rituximab-chlorambucil. For fit and young patients (<60–65 years with no significant comorbidities), current evidence suggests that optimal treatment is intensive chemotherapy with a view to achievinga complete response prior to autologous peripheral blood stem cell transplantation. Suitable regimens would include rituximab and high-dose cytarabine.^[16]^

Bullous pemphigoid associated with mantle cell lymphoma is very rare with only one case has been reported in the literature.^[1]^ The previous reported case was a young 39-yearold Caucasian man who presented with severe odynophagia for 3 months resulting in weight loss of 5 kg and extensive eruption of tense blisters and painful oral lesions for 6 weeks. The patient managed to undergo an allogeneic bone marrow transplantation after cytoreduction with 2 courses of fractionated cyclophosphamide, doxorubicin hydrochloride, vincristine sulfate, and dexamethasone, which were alternated with high-dose methotrexate and cytosine arabinoside after the diagnosis of mantle cell lymphoma stage IVa was established. The bullous pemphigoid lesions gradually disappeared during cytoreduction and remain stable until after 48 months of follow-up, and required no further topical or systemic therapy. In our case, the presentation of bullous pemphigoid led to the detection of mantle cell lymphoma. However, the late presentation at diagnosis and aggressiveness of the disease, patient’s condition had deteriorated after commencing cytoreduction chemotherapy. Both, bullous pemphigoid and mantle cell lymphoma had a parallel clinical course which suggests a paraneoplastic phenomenon in this reported case.

## 4. Conclusion

Bullous pemphigoid associated with mantle cell lymphoma are very rare as seen in our case report. The presentation of bullous pemphigoid led to the detection of mantle cell lymphoma. Early detection of the diseases and appropriate multi-discipline team approach is essential in managing this type of the disease.

## Acknowledgments

The authors would like to acknowledge Universiti Teknologi MARA (UiTM) Sungai Buloh for supporting the submission of the following article.

## Author contributions

**Writing – original draft:** Ahmad Bakhtiar Md Radzi.

**Writing – review & editing:** Sazzli Shahlan Kasim, Ahmad Bakhtiar Md Radzi.
